# Supplementary material for: Bayesian refinement of protein structures and ensembles against SAXS data using molecular dynamics
Source: PLoS Comput Biol. 2017 Oct 18;13(10):e1005800. doi: 10.1371/journal.pcbi.1005800 (PMC5662244; doi:10.1371/journal.pcbi.1005800)
Supplement: S1 Archive — (BZ2) [file pcbi.1005800.s011.tar.bz2 › gromacs_4.62_bayesian_saxs_refi/share/html/online/dlg.html]

dlg file format


## dlg file format

|  |  |
| --- | --- |
| Main Table of Contents | **VERSION 4.6** |
| GROMACS homepage | **Sat 19 Jan 2013** |

---

### Description

The dlg file format is used as input for the ngmx
trajectory viewer. These files are not meant to be altered bu the end user.

### Sample

```
	
grid 39 18 {

group "Bond Options" 1 1 16 9 {
  radiobuttons { " Thin Bonds"  " Fat Bonds" " Very Fat Bonds" " Spheres" }
	"bonds" "Ok" " F" "help bonds"
}	

group "Other Options" 18 1 20 13 {
  checkbox " Show Hydrogens"  ""   	"" "FALSE"	"help opts"
  checkbox " Draw plus for atoms" ""    "" "TRUE"       "help opts"
  checkbox " Show Box"		""	"" "TRUE"	"help opts"
  checkbox " Remove PBC"	""	"" "FALSE"	"help opts"
  checkbox " Depth Cueing"	""	"" "TRUE"	"help opts"
  edittext "Skip frames: "	""	"" "0"		"help opts"
}

simple 1 15 37 2 {
  defbutton "Ok" "Ok" "Ok" "Ok" "help bonds"
}

}
```

---

http://www.gromacs.org
